# Metagenomic sequencing for investigation of a national keratoconjunctivitis outbreak, Israel, 2022

**DOI:** 10.2807/1560-7917.ES.2023.28.31.2300010

**Published:** 2023-08-03

**Authors:** Yair Motro, Denise Wajnsztajn, Ayelet Michael-Gayego, Shubham Mathur, Roberto BM Marano, Ikram Salah, Chaggai Rosenbluh, Violeta Temper, Jacob Strahilevitz, Jacob Moran-Gilad

**Affiliations:** 1 Department of Health Policy and Management, School of Public Health, Faculty of Health Sciences, Ben Gurion University of the Negev, Beer Sheva, Israel; 2 Department of Ophthalmology, Hadassah Hebrew University Medical Center, Jerusalem, Israel; 3 Clinical Microbiology Laboratory, Department of Clinical Microbiology and Infectious Diseases, Hadassah Hebrew University Medical Center, Jerusalem, Israel; 4 Department of Genetics, Hadassah Hebrew University Medical Center, Jerusalem, Israel; * These authors contributed equally to the manuscript and share first authorship

**Keywords:** keratoconjunctivitis, *Microsporidium*, PCR, metagenomics, outbreak investigation, unknown aetiology, public health microbiology

## Abstract

**Background:**

Epidemics of keratoconjunctivitis may involve various aetiological agents. Microsporidia are an uncommon difficult-to-diagnose cause of such outbreaks.

**Aim:**

During the third quarter of 2022, a keratoconjunctivitis outbreak was reported across Israel, related to common water exposure to the Sea of Galilee. We report a comprehensive diagnostic approach that identified *Vittaforma corneae* as the aetiology, serving as proof of concept for using real-time metagenomics for outbreak investigation.

**Methods:**

Corneal scraping samples from a clinical case were subjected to standard microbiological testing. Samples were tested by calcofluor white staining and metagenomic short-read sequencing. We analysed the metagenome for taxonomical assignment and isolation of metagenome-assembled genome (MAG). Targets for a novel PCR were identified, and the assay was applied to clinical and environmental samples and confirmed by long-read metagenomic sequencing.

**Results:**

Fluorescent microscopy was suggestive of microsporidiosis. The most abundant species (96.5%) on metagenomics analysis was *V. corneae*. Annotation of the MAG confirmed the species assignment. A unique PCR target in the microsporidian rRNA gene was identified and validated against the clinical sample. The assay and metagenomic sequencing confirmed *V. corneae* in an environmental sludge sample collected at the exposure site.

**Conclusions:**

The real-time utilisation of metagenomics allowed species detection and development of diagnostic tools, which aided in outbreak source tracking and can be applied for future cases. Metagenomics allows a fully culture-independent investigation and is an important modality for public health microbiology.

**Key public health message**

**What did you want to address in this study?**

We wanted to use advanced diagnostic methods to identify the cause of an elusive national outbreak of keratoconjunctivitis (acute eye infection) and to develop diagnostic tools that can be quickly adapted to a new outbreak investigation.

**What have we learnt from this study?**

With metagenomic next-generation sequencing and a novel real-time PCR assay, we established that exposure to freshwater containing *Vittaforma corneae* was the likely source of the keratoconjunctivitis outbreak.

**What are the implications of your findings for public health?**

So far, this sequencing method has mainly been applied for difficult-to-diagnose clinical cases. Our study provides proof-of-concept that this approach can be used to identify the source of an outbreak in real time and to develop outbreak investigation tools ad hoc.

## Introduction

Keratoconjunctivitis is an acute eye infection implicating a wide range of causative pathogens, mainly bacteria and viruses (including staphylococci, *Pseudomonas aeruginosa* and adenovirus) [1]. Other causes of keratoconjunctivitis include parasites, especially free-living amoeba [2], and fungal pathogens such as *Fusarium* and *Aspergillus* [3]. All are readily identified by standard laboratory methods.

Keratoconjunctivitis may be associated with various risk factors, including environmental exposure, eye trauma or surgery, underlying ocular surface disease and use of contact lenses [1]. Epidemics of keratoconjunctivitis can occur and may involve seasonal activity of viruses such as adenovirus [4] or factors affecting amoebal activity such as hot weather, water quality and natural disasters [2].

Microsporidia are ubiquitous degenerate fungi that can cause a wide spectrum of infections in immunocompromised and immunocompetent hosts [5], affecting multiple organs such as the gastrointestinal tract, musculoskeletal system and central nervous system. Reports of microsporidial keratoconjunctivitis have increased in recent years, affecting patients across all age groups, usually with a satisfactory outcome [6]. Whether this increase in incidence is related to changing epidemiology or improved diagnosis is still debated. Because microsporidia are highly ubiquitous in natural environments, identifying an epidemiological link between an infecting species and an environmental source is essential for implementing effective control strategies [7].

Multiple *Micorsporidium* species have been implicated in sporadic and epidemic keratoconjunctivitis in immunocompetent hosts, especially *Encephalitozoon hellem* and *Vittaforma corneae* (previously *Nosema corneum*) [5]. The main diagnostic procedures for detecting ocular microsporidiosis are microscopy and PCR-based techniques. The accuracy of PCR is limited in light of the different *Microsporidium* species implicated in keratoconjunctivitis, and thus the definitive identification of the microsporidia at the species level remains challenging. More recently, metagenomic next-generation sequencing (mNGS) enabled the post-mortem identification of an invasive *Anncaliia algerae* infection in an immunosuppressed kidney transplant recipient after direct PCR failed to detect the microsporidial infection [8].

We report a comprehensive diagnostic approach, including mNGS and ad hoc PCR development and application, which enabled the diagnosis of *V. corneae* infection in a clinical case of keratoconjunctivitis, as part of a national outbreak of unknown aetiology, and identification of the likely water source of this infection.

## Case description

Between August and November 2022, a large number of cases of keratoconjunctivitis were reported across Israel. All patients appeared to have had recreational water exposure in the Sea of Galilee in Northern Israel (Lake Kineret), a popular resort. It is estimated that at least 40 cases have been affected [9]. Standard microbiological investigations (Gram stain and culture) in different institutions failed to detect the aetiology of the outbreak.

**Figure 1:**
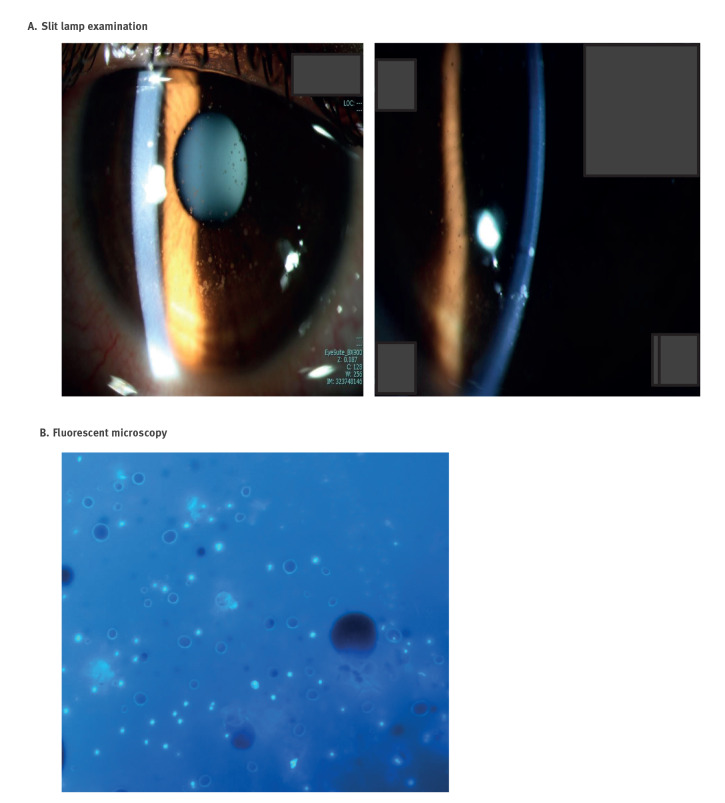
Slit lamp and fluorescent microscopy findings, keratoconjunctivitis patient, Israel, 2022

In November 2022, an 18-year-old patient (Jerusalem, Israel) presented with left eye hyperaemia and discomfort for the past 10 days. Fourteen days before the start of symptoms, they swam in the Sea of Galilee. At presentation, the visual acuity was reduced, and they had conjunctival hyperaemia and multiple and diffuse superficial round epithelial and intra-epithelial opacities (Figure 1A). A large 8.0 mm epithelial debridement was performed, and samples were submitted for microbiological investigation. After 4 days, there was a complete epithelialisation, and 11 days post-debridement, the patient was asymptomatic. During follow-up, a clear cornea was evident, with some fading subepithelial haze and no corneal staining, and their vision improved.

Panel A: Slit lamp examination of the left eye with indirect illumination showing diffuse superficial corneal round opacities (left picture) and the subepithelial location of round corneal opacities (right picture).

Panel B: Fluorescent microscopy of calcofluor white stained preparation of scraped corneal material showing round white homogenous structures compatible with microsporidia. The slide was visualised with a 420 nm filter at 365 nm excitation and 395 nm emission.

## Methods

### Routine microbiological work-up

Corneal scraping material was subjected to standard microbiological work-up at the Clinical Microbiology Laboratory of Hadassah Medical Center, including Gram stain and microscopy, and attempted bacterial and fungal culture. We extracted DNA using the QIAamp DNA Mini Kit following pre-treatment with ATL buffer and proteinase K (QIAGEN) and performed in-house PCR assays for *Acanthamoeba* spp. as well as broad-range PCRs targeting the 16S ribosomal RNA (rRNA) gene (pan-bacterial assay) and the internal transcribed spacer of the rRNA gene (pan-fungal assay). Microscopy for suspected microsporidian infection was performed on corneal scraping material using Calcofluor white staining, using 30 µL of 0.1% Fluorescent Brightener 28 mixed with 10% KOH (Sigma-Aldrich). The slide was visualised under a fluorescent Axioskop microscope (Zeiss) with a 420 nm filter at 365 nm excitation and 395 nm emission.

### Clinical metagenomic sequencing

We re-extracted the corneal sample using the above method, yielding 5.8 ng/µL of DNA. Whole genome sequencing was performed at the NGS Unit of the Department of Genetics, Hadassah Medical Center. Sequencing libraries were prepared from 500 ng of DNA with the Illumina Nextera Flex Library Prep Kit and sequenced on the Illumina NovaSeq6000 instrument. Sequencing was performed on an S2–300 flowcell yielding a total of 129,074,310 raw reads.

### Bioinformatics analysis

Short sequence reads underwent quality control (using fastp, v0.23.2) and taxonomic assignment using kraken2 (v2.0.8) with the Standard-8, Viral and EuPathDB46 databases. The latter includes nearly 400 genomes of eukaryotic pathogens, including microsporidia (available at http://ccb.jhu.edu/data/eupathDB). We removed host (human) reads using kraken2 assignment with the Standard-8 database and KrakenTools (v1.2, extract_kraken_reads.py with parameters ‘--taxid 9606 --exclude’). To assess possible spurious taxonomical assignments, we mapped extracted reads of the taxa relatively abundant in the sample (> 100 reads) against reference genomes of the respective taxa and measured the breadth of coverage was using minimap2 (v2.22-r1101) and samtools (v1.9 with htslib v1.9).

Unclassified reads were subjected to additional analysis at protein level using mmseqs2 (v 14.7e284). The UniRef50 database (https://www.uniprot.org/help/uniref) was downloaded using the mmseqs ‘databases’ command. An mmseqs database was then created with the unclassified reads using the mmseqs ‘createdb’ command and was annotated via similarity search against the UniRef50 database using the mmseqs ‘taxonomy’ command (with the parameter ‘-s 2’). Taxonomic assignment results were summarised using the mmseqs ‘createtsv’ and ‘taxonomyreport’ commands.

Host-filtered reads (ca 1.8 million paired reads) were de novo assembled using Megahit (v1.2.9), and taxonomic assignments of the contigs were assigned using kraken2 with the EuPathDB46 database to identify potential eukaryotic pathogen sequences. Contigs identified as *V. corneae* in the previous step were further selected for analyses. We compared the resultant metagenome-assembled genome (MAG) with the reference genome of *V. corneae* ATCC 50505 (National Center for Biotechnology Information (NCBI) GenBank accession GCA_000231115.1) using progressiveMauve (v2.4.0) and mummer (v3.1). The isolated MAG contigs were arranged according to the reference genome of *V. corneae* ATCC 50505 contigs using the tool jContigSort (v1.3). Plots were generated using mummer2circos (v1.4.2) and pg-mauve (v0.3.1). Depth of coverage of the isolated MAG was calculated using minimap2 (v2.22-r1101) and samtools (v1.9 with htslib v1.9).

After annotating the isolated MAG with Prokka (v1.14.5), species assignment of *V. corneae* was confirmed by comparing the predicted small-subunit (SSU) 18S rRNA gene to the NCBI nucleotide database using megaBLAST. A multiple sequence alignment (using mafft, v7.475 and trimAl, v1.4) was conducted against *V. corneae* SSU 18S rRNA gene sequences obtained from the SILVA database (r138.1) and the MAG’s 18S rRNA gene, and a subsequent maximum-likelihood (ML) phylogenetic tree was built using IQ-Tree2 (v2.1.2).

### Real-time PCR development and validation

We performed in silico PCR on the isolated MAG and the *V. corneae* ATCC 50505 reference genome using primer sequences available from the literature (Table) with seqkit (v2.3.0, using the ‘amplicon’ subcommand). An amplicon was generated from both the isolated MAG and reference genomes using the primer set LPW20469-LPW20470 while other primer sets failed (Table). Since the former primers are pan-microsporidian, we sought to utilise a *V. corneae*-specific primer pair. Upon close inspection of the failed primers LPW20475-LPW20476, we found a number of base variations in the MAG primer binding sites. We therefore redesigned the primers LPW20475-LPW20476 by selecting a conserved downstream region with Primer3 (using the ‘check primers’ module with the isolated MAG 18S rRNA gene as template sequence). The optimised primers (Forward -5’-GGTTGACGGGAAATTAGGGT-3’; Tm = 57.5 °C; Reverse -5’-CTGCAGCATCTCTCATACACAC-3’; Tm = 59.1 °C) were confirmed in silico against the MAG and then tested in vitro using an ad hoc SYBR green-based assay. The full real-time PCR protocol can be accessed in the Supplement. The PCR products were Sanger sequenced and analysed against the NCBI nucleotide database using megaBLAST.

**Table:** Primers used for in silico PCR development and validation, keratoconjunctivitis outbreak, Israel, 2022

Primer specificity	Primer set label	Forward (5’ → 3’)	Reverse (5’ → 3’)	Amplicon size (bases)	Reference
Pan-microsporidial	LPW20469-LPW20470	CACCAGGTTGATTCTGCC	GTGACGGGCGGTGTGTAC	1,200	[27]
	Microspo	TTCCGGAGAGGGAGCCTGAGAG	CCACTCCTTGTGGTGTCCTTCCGTCAA	558	[13]
*Vittaforma corneae* species- specific	Vc-specific	CTACCAAGACAGTGACGGTTGA	GGCATCTTTTACTGCTGGAACT	472	[14]
	LPW20475-LPW20476	ACAGTGACGGTTGACGGGAA	TGAGACCTCGCATCTCTCTTT	280	[27]
	HMC-Vc	GGTTGACGGGAAATTAGGGT	CTGCAGCATCTCTCATACACAC	224	This study

### Environmental sampling, molecular testing and metagenomic sequencing

To identify a potential environmental source, three freshwater (à 1,000 mL) and three underwater sludge samples (à 50 mL) were obtained about 6 weeks following clinical presentation, from the specific coastline to which the patient reported exposure. Sampling involved three beach locations along a continuous coastline on a single day. Samples were processed following vigorous shaking and filtration of 20 mL using a 0.45 μm filter, followed by extraction using the DNeasy PowerWater kit (QIAGEN) per manufacturer’s instructions. DNA extracts were then tested using the novel real-time PCR (HMC-Vc) and amplicons were Sanger-sequenced for confirmation. Sequence traces showing double peaks underwent deconvolution using Tracy (v0.7.3), using the ‘decompose’ subcommand using the isolated MAG’s 18S rRNA gene as reference. An in-house 16S rRNA broad-range TaqMan qPCR assay was performed in parallel as process control.

Positive samples were further analysed using long-read metagenomic sequencing. Briefly, ca 1 µg of DNA was prepared with the SQK-LSK109 Ligation Sequencing Kit (ONT). Sequencing was carried out using the Minion Oxford Nanopore (ONT) with an R9.4.1 Flow cell (FLO-MIN106). Long reads were basecalled using Guppy (v6.0.1 + 652ffd1, with the HAC mode, and parameters ‘--trim_adapters --kit SQK-LSK109 --flowcell FLO-MIN106’). Reads longer than 1 kb were analysed using kraken2 (v2.0.8) with the Standard-8, Viral and EuPathDB46 databases, as above, to identify potential pathogens. Reads that were assigned to the phylum Microsporidia (taxid 6029) were filtered using KrakenTools (v1.2, extract_kraken_reads.py with parameters ‘--taxid 6029 --include-children’) and compared with the isolated MAG and *V. corneae* ATCC 50505 reference genome using BLASTn. To explore strain-level relatedness of environmental *V. corneae* reads, host-removed short reads of the clinical metagenome described above were mapped to all long reads that were assigned to the taxon *V. corneae* (taxid 42399) using minimap2 and samtools. The mapped reads were analysed for breadth and depth of coverage.

### Analysis of historical samples from the Sea of Galilee

We downloaded a publicly available metagenomic sequencing data of 61 samples from the Sea of Galilee from the NCBI SRA database (https://www.ncbi.nlm.nih.gov/sra; accessed in December 2022), under the bioprojects PRJNA488159, PRJNA497963 and PRJNA637457. Taxonomic assignments of each sample’s reads were performed using kraken2 with the EuPathDB46 database to identify potential eukaryotic pathogen sequences, and summarised using KrakenTools (v1.2, combine_kreports.py). Reads that were assigned to the taxon *V. corneae* ATCC 50505 (taxid 993615) for each sample were mapped to the isolated MAG using minimap2. For the samples harbouring the greatest number of reads assigned to *V. Corneae* (SRR8088693, SRR7764488, SRR7764487, and SRR7764492), reads were mapped to the SSU of the isolated MAG. Consensus sequences were generated from these samples using samtools consensus and aligned to the amplicons generated in silico from the optimised primers as described above.

## Results

### Routine microbiological analyses

Routine microscopy and bacterial and fungal culture were unremarkable, yielding no pathogen. The broad-range PCR assays and the specific PCR for free-living amoeba were also negative. Calcofluor White staining analysed by fluorescent microscopy demonstrated small round fluorescent cells suggestive of *Microsporidium* cells (Figure 1B).

### Metagenomic sequencing of the clinical sample

Sequencing of the mNGS short reads (Illumina) produced 129 million high-quality paired-end reads with an average size of 147 bp. Taxonomic assignment of the mNGS reads assigned 86.12% of the reads to human origin, while 0.01% were assigned to either bacterial or viral sequences, and the remaining 13.87% were unassigned; for the exact number of reads see Supplementary Table S1. Assigned bacterial sequences included 3,759 taxa of which only several taxa were assigned more than 50 reads. When mapped against the reference genomes of the respective genera (*Salmonella*, *Bartonella*, *Klebsiella*, *Streptomyces*, *Bacillus*), the reads covered only a small fraction of their genomes (between 0 and 2,000 bases), suggesting spurious assignments. Analysis against the Virus database did not reveal any pathogenic viruses; a list of these viruses is appended in Supplementary Table S2.

After removal of the host (human) reads, we compared the remaining 3.7 million reads with the eukaryotic pathogens database (EuPathDB46) using kraken2, where 96.5% of the assigned reads (34,665/35,895) matched to *V. corneae*, thus establishing the diagnosis of ocular microsporidiosis; we provide a list of the matched taxa in Supplementary Table S3. Another 396 reads were assigned to several other closely related microsporidia while 834 reads were assigned to 19 other eukaryotic taxa at negligible abundance (20–65 reads each), probably representing misclassified *V. corneae* reads.

We subsequently analysed the 3,650,413 unclassified reads at protein level. Of these, 1,921,058 reads passed the mmseqs pre-filtering steps, while 1,031,665 reads remained unclassified. An additional 90,654 reads were ambiguously assigned as either root or cellular organisms. Of the classified reads, the most abundant taxonomic assignment was *V. corneae* ATCC 50505 (515,515 reads; 26.8%) or other microsporidia at different taxonomical levels (69,389 reads; 3.6%). The remaining reads were assigned to several thousand different taxa across different kingdoms, at abundancies of less than 0.1%.

**Figure 2:**
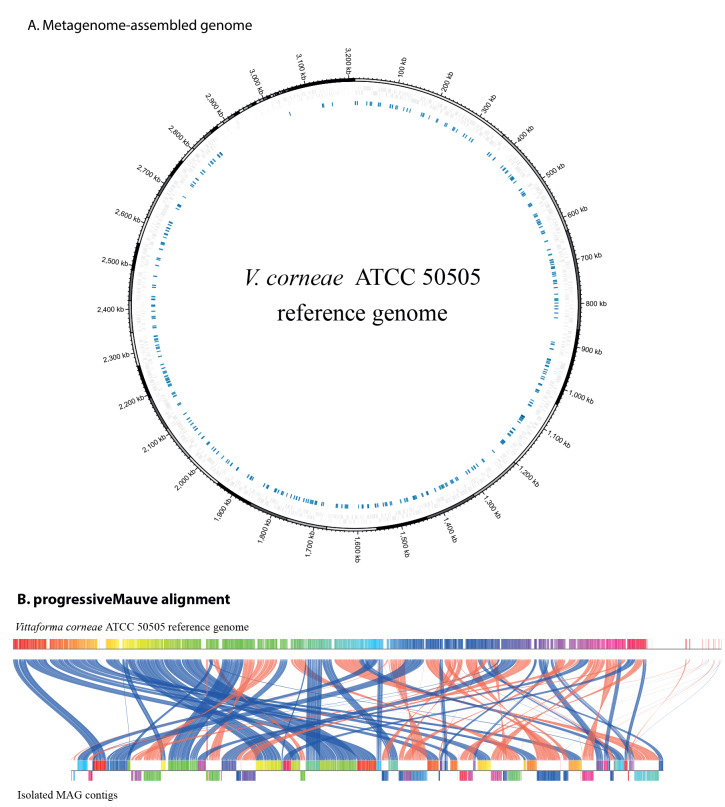
Comparison of the isolated MAG contigs with the *Vittaforma corneae* ATCC 50505 reference genome, keratoconjunctivitis patient, Israel, 2022

ATCC: American Type Culture Collection; MAG: metagenome-assembled genome.

The host (human)-removed mNGS reads underwent de novo assembly, generating a 3.6 Mb metagenomic assembly consisting of 1,645 contigs (N50 47,773). Taxonomic assignments of the metagenomic assembly contigs to the eukaryotic pathogens database (EuPathDB46) using kraken2 assigned 69 contigs to *V. corneae*, totalling 2.67 Mb, with a 61-fold mean depth of coverage and a contiguity of N50 of 64,665. Comparison of the isolated MAG against the *V. corneae* ATCC 50505 reference genome showed 71.3% breadth of coverage (Figure 2). After filtering the complete metagenome assembly by size, only 116 of the unassigned contigs (7.3%) were > 1 kb. Repeated analysis classified one contig as a related microsporidium (*Enterocytozoon bieneusi* H348) and another 107 contigs as eukaryotic or root or cellular organism.

Panel A: The outer ring (contigs coloured black, grey or white) of the mummer2circos plot shows the *V. corneae* ATCC 50505 reference genome sequence, the middle ring (grey) shows the coding regions in both directions in the reference genome, the innermost ring (blue) shows the isolated MAG’s contigs that aligned to the reference genome (using mummer).

Panel B: progressiveMauve alignment between the *V. corneae* ATCC 50505 reference genome (top) and the isolated MAG contigs (bottom). Aligned contigs are coloured according to their arrangement in the reference genome. Connecting lines (coloured blue for same direction alignment, or orange for opposing direction alignment) between the reference genome and the isolated MAG contigs show the aligned regions.

**Figure 3:**
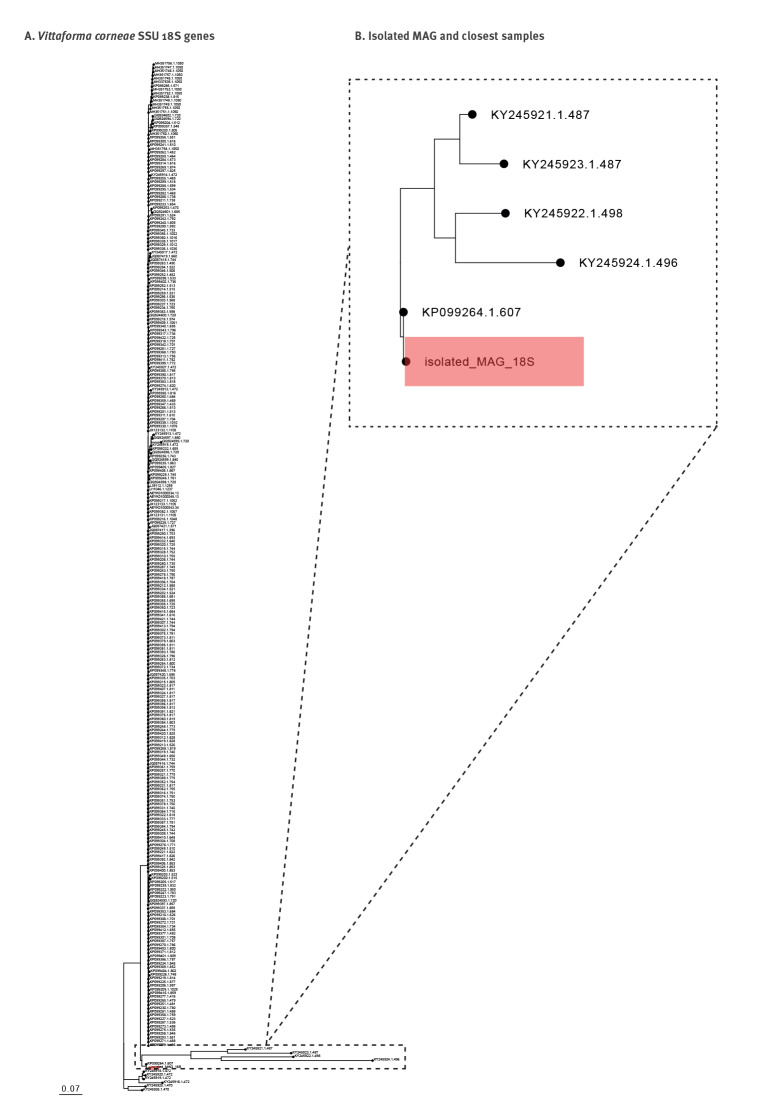
Phylogenetic analysis of *Vittaforma corneae* from a keratoconjunctivitis patient, Israel, 2022

MAG: metagenome-assembled genome; ML: maximum likelihood; SSU: small subunit.

The isolated MAG’s annotated 18S rRNA gene was aligned against 278 *V. corneae* SSU 18S rRNA genes obtained from the SILVA rRNA database. The derived ML phylogenetic tree (Figure 3) shows the isolated MAG clustered outside of the main cluster of *V. corneae* samples obtained from the SILVA database. The nearest sample to the isolated MAG (KP099264.1.607) involved a human ocular sample from India, dated 2015, at 99.5% identity.

Panel A: ML phylogenetic tree based on the SSU 18S rRNA gene from the isolated MAG (highlighted in red) and 278 *V. corneae* SSU 18S genes obtained from the SILVA database.

Panel B: Enlargement of the isolated MAG and closest samples in the phylogenetic tree. The nearest sample to the isolated MAG (KP099264.1.607) involved a human ocular sample from India, dated 2015.

### Molecular analysis of the clinical sample

**Figure 4:**
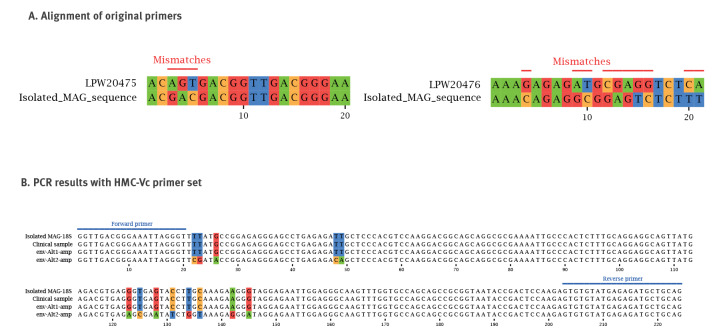
Development and application of real-time PCR for *Vittaforma corneae*, Israel, 2022

Due to a number of base variations in the isolated MAG primer binding sites (Figure 4A), we redesigned and optimised the *V. corneae*-specific primers (LPW20475-LPW20476 primer set). The new primers (HMC-Vc, Table) were applied in triplicate on a DNA extract from the original corneal scraping material and yielded a clear positive result with a melting temperature of 85.8 °C. The PCR product was subjected to Sanger sequencing and demonstrated 100% identity to the predicted MAG-derived amplicon and 96.43% identity to *V. corneae* deposited in NCBI.

MAG: metagenome-assembled genome.

Panel A: LPW20475-LPW20476 PCR primer set (see Table) alignment to isolated MAG sequence for mismatch identification. Several forward and reverse base variations, necessitating optimisation, are highlighted.

Panel B: Optimised PCR primer set testing results, with the forward and reverse primers binding sequences highlighted in blue. PCR testing of the clinical sample (second row) produced an amplicon with 100% match to the 18S rRNA MAG target (first row). PCR testing of the environmental sample produced two potential amplicons (after deconvolution of trace files): Alt1-amp (third row) showed an exact match to the 18S rRNA MAG target, while Alt2-amp (fourth row) showed 13 mismatches.

### Molecular and metagenomic analysis of environmental samples

We tested six environmental samples; three samples contained 1,000 mL of freshwater, whereas three others contained ca 50 mL of underwater solids (sludge, pebbles and gravel) mixed in freshwater from each of the sampled sites. All samples were positive for 16S rRNA in the qPCR performed as process control, reflecting a concentration of ca 10^9^ bacterial cfu/L. One of the six samples was positive for *V*. *corneae* by means of the novel real-time PCR. Sanger sequencing of the PCR product yielded the expected amplicon size (224 bases) with multiple evident double peaks, suggesting a possible mixture of several microsporidian amplicons. The Sanger sequences were deconvoluted, producing two possible closely related amplicon sequences (Figure 4B). The two amplicon sequences were aligned with the MAG, showing 100% (Alt1-amp) and 94.2% (Alt2-amp) identity. We also queried the deconvoluted amplicons against the NCBI nucleotide database using megaBLAST, with top matches being *V. corneae* and *Microsporidium* sp., respectively; the BLAST search details can be accessed in Supplementary Table S4.

Long read sequencing produced 1.42 million reads with an average size of 4,536 bases (range: 1,000–77,496). Taxonomic assignment using kraken2 identified human (4.71%), archaeal (0.19%), bacterial (30.78%) and viral (0.03%) sequences; we refer to Supplementary Table S5 for the exact numbers. We also compared the long reads with the eukaryotic pathogens database (EuPathDB46) using kraken2, with 11,992 reads (0.84%) assigned a phylum (listed in Supplementary Table S6), of which 117 reads (ranging between 1,088 and 37,669 bases in size) were assigned to microsporidia. These included three long reads assigned to *V. corneae* (17,485, 6,710 and 6,671 bases in size) and other reads assigned to non-*Vittaforma* species; for the complete list see Supplementary Table S7. This was consistent with the PCR results, indicating the presence of several microsporidian species in the sample and the presence of *V. corneae* at the site of reported exposure. Analysis using the kraken Virus database revealed non-pathogenic viruses and analysis for bacteria revealed mostly environmental taxa but also several species of enteric bacteria at different quantities; these species and read numbers can be accessed in Supplementary Tables S8 and S9.

### Strain-level analysis of *Vittaforma corneae* from short and long reads

**Figure 5:**
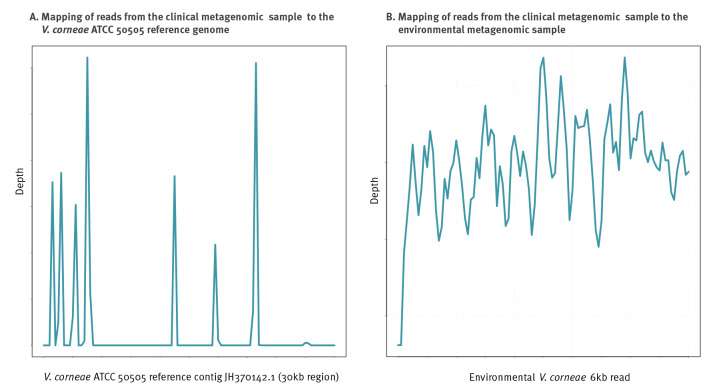
Mapping of clinical metagenomic sample to environmental metagenomic sample and to *Vittaforma corneae* ATCC 50505, keratoconjunctivitis patient, Israel, 2022

We attempted mapping the host-removed mNGS short reads from the clinical sample to each of the three *V. corneae*-assigned long reads (3.7 million reads, of which 1,131,712 mapped to the MAG). This resulted in mapping of 2,798 short reads to one long read (6,671 bases in size), with 98.91% breadth of coverage and 59.91-fold depth of coverage. When these short reads were mapped to the *V. corneae* ATCC 50505 reference genome, only 2,239 bases in a 30,000-base region in contig JH370142.1 were covered, with 7.46% breadth of coverage and 1.97-fold mean depth of coverage (Figure 5); details are appended in Supplementary Table S10.

ATCC: American Type Culture Collection.

Host-removed reads from clinical metagenomic sample (3.7 million) covered 2,239 bases in a 30 kb region of the *V. corneae* ATCC 50505 contig JH370142.1 (at a mean depth of 2 ×) and 6,598 bases (at a mean depth of 60 ×) in the environmental 6 kb *V. corneae* read.

### Analysis of historical metagenomes from the Sea of Galilee

We analysed a total of 61 metagenomic short reads from samples taken between 2013 and 2017 in the Sea of Galilee, totalling 1,459,047,627 reads (mean: 23,918,814, median: 21,977,956; range: 401,405–510,04,644). After taxonomic assignment of the reads using kraken2 with the EuPathDB46 database, 27,198 reads were assigned to the phylum Microsporidia, of which 460 reads were assigned to the species *V. corneae*. The distribution of *V. corneae* reads across historical samples is appended in Supplementary Figure S1. For each of the four most abundant samples, mapping of the reads to the MAG provided a consensus sequence for the SSU. When aligned to our 224 bp PCR amplicon (including the isolated MAG, reference genome and environmental sample amplicons as shown in Figure 4B), regions of variation were observed for all samples; the alignment for these is appended in Supplementary Figure S2.

## Discussion

We describe the establishment of an aetiological diagnosis and attempted source attribution of a nationwide keratoconjunctivitis outbreak using mNGS and development of complementary molecular diagnostics. The aetiology of this outbreak remained unknown for several months; therefore, the confirmation of *V. corneae*, a microsporidian pathogen known to cause keratoconjunctivitis, had an important public health impact. Following the confirmation of the case reported herein, several cases have been reported as confirmed *V. corneae* infection using NGS or PCR elsewhere in Israel [10]. 

The main clinical manifestations of microsporidiosis among immunocompetent patients are usually self-limited gastroenteritis and keratoconjunctivitis, and *V. corneae* is a predominant aetiology of the latter, causing either sporadic cases or outbreaks. Nine cases were reported in Taiwan in 2011 and linked to two types of non-disinfected hot springs [11]. Another outbreak in Taiwan in 2017 involved 13 teenagers following exposure to a local swimming pool [12]. Another outbreak was reported from Singapore following a rugby tournament; it involved 47 cases, attributed to exposure to muddy field conditions following local floods [7]. Interestingly, only five cases in that outbreak were microbiologically confirmed, and the authors mention that liberal corneal scraping could not be justified.

There is a wide range of pathogens that could be considered as difficult to diagnose. Such pathogens may include unculturable microorganisms (e.g. *Tropheryma whipplei* or rickettsial organisms), organisms that are very difficult to culture such a spirochetes (e.g. *Leptospira* or *Borrelia*) or organisms for which routine diagnostics such as validated PCR assays are not widely available. The epidemiology of such pathogens and their scarcity often results in a lack of developed commercial or in-house diagnostic solutions. Microsporidia can be considered such difficult-to-diagnose organisms. In addition, obtaining high-quality sample material might not be trivial for some types of infection and can become a limiting factor. In that respect, corneal scrapings could be considered such samples that may not be widely available. Our case confirms the usefulness of fluorescent microscopy in diagnosing ocular microsporidiosis in cases where corneal samples are obtained. Nevertheless, expertise in microscopic diagnosis of microsporidia is lacking in many laboratories. Moreover, in the context of an elusive outbreak, microscopy may be insufficient for establishing the aetiology for a sentinel case but could be used in further cases once the aetiology has been unequivocally confirmed using molecular or genomic tools.

Several diagnostic PCR assays for *Microsporidium* detection in keratoconjunctivitis have been published, primarily targeting the SSU ribosomal 18S rRNA gene [13]. Another approach is a dual PCR assay comprised of SSU PCR as a target for microsporidia detection and a complementary target specific to *Vittaforma* [14]. Due to the notable diversity of *Microsporidium* spp., the accuracy of PCR in this setting is far from optimal, creating the need for a more comprehensive approach, such as mNGS.

Use of mNGS is increasingly being reported in clinical microbiology as a cutting-edge diagnostic modality [15] having impact on the diagnosis of infections across all organ systems, such as the central nervous, musculoskeletal or respiratory system infections. mNGS has been used successfully for the diagnosis of serious eye infection such as ocular Whipple’s disease [16], bacterial endophthalmitis [17,18] and uveitis [19] but also of more superficial eye infections such as acute conjunctivitis, infectious keratitis and corneal ulcers [20-22]. Several challenges still surround the application of mNGS in the diagnosis of eye infection, including the order by which samples are obtained [23], difficulties in validation and interpretation, the limit of detection, reagent contamination, regulatory aspects and costs, to name a few [24]. While the reports of mNGS in ophthalmology have not focused on microsporidiosis, a recent report from China describes an invasive fatal microsporidial infection in which *A. algerae* was detected by deep sequencing of respiratory and muscle tissue samples [8]. Light and transmission electron microscopy yielded compatible findings. This report exemplifies the utility of mNGS in the diagnosis of elusive cases.

Waterborne microsporidiosis can be linked to a putative source of exposure based on conventional epidemiological methods elucidating behavioural risk factors. Nevertheless, microbiological confirmation of an outbreak source may be challenging. Based on PCR testing of water and soil samples, a follow-up study of the 2017 Taiwan outbreak has stipulated that environmental contamination following rainfall resulted in secondary swimming pool contamination following human activity [25]. Notably, sequencing of PCR products revealed a high sequence diversity with only a few amplicons being similar to those derived from clinical samples, suggesting that environmental contamination with microsporidia involved multiple clones. In a further ecological study, molecular surveillance of *Vittaforma*-like organisms in rivers and water reservoirs in Taiwan identified positive samples in 47 of 50 tested sites [26]. That study also reported a notable phylogenetic diversity based on sequenced PCR amplicons, with only 32 of 82 amplicons analysed showing a very close identity to *V. corneae*. Moreover, most samples yielded two or three bands on DNA electrophoresis, suggesting a mixture of different microsporidia, a finding confirmed by cloning and aligning sequences. These environmental findings coincide with our real-time PCR results, which showed evidence of two microsporidia in the environmental samples, one being *V. corneae* identical to the clinical sample and the other a closely related unidentified *Microsporidium sp*. Furthermore, our analysis of historical samples also confirmed the abundance of different microsporidia in the Sea of Galilee, including different strains of *V. corneae*, which were closely related but not identical to the outbreak strain, testifying to the complexity of this ecosystem.

The long-read metagenomic sequencing results further supported this finding. Sequencing of the environmental sample identified several microsporidia co-existing in the sample. While a complete strain-level metagenomic analysis could not be carried out, due to the paucity of public genomes and the small number of high-quality long reads identified, mapping of the short reads of the clinical sample to a 6 kb read, resulted in very high depth and coverage, a finding highly suggestive of the infection source in the environmental sample.

Use of mNGS enables a range of microbiological applications in a single test, including the detection of known pathogens in an unbiased manner (as opposed to the hypothesis-driven utilisation of PCR), the study of microbial populations (such as the microbiome) or pathogen discovery in case of an emerging or new pathogens. With the appropriate computational approach, mNGS could also allow interrogation of identified genomes within a metagenome to perform strain-level analysis (e.g. for resistance and virulence determinants), determination of phylogeny, and identification of diagnostic targets for specific PCR development. In the context of an outbreak lacking a clear aetiology, mNGS is especially powerful since it allows relatively rapid culture-independent detection of the causative pathogens while facilitating comparison to previous or other cases and the ad hoc development of diagnostics, which could enable further case finding or source tracking. Notably, in the case presented herein, mNGS was also useful in searching for alternative aetiologies such as common bacterial and viral causes of keratoconjunctivitis, further supporting the established diagnosis.

Our study has several limitations. Corneal samples from additional patients were not available and therefore a strain-level comparison of *V. corneae* between outbreak cases was not possible. Moreover, in the absence of additional samples, clinical validation of the ad hoc PCR assay could not be performed beyond the current case. The depth of long-read sequencing of the environmental sample was limited, thus not allowing a complete comparison of the MAG between the short-reads and long-reads metagenome. Despite using several analytical tools, many sequencing reads remained unclassified or resulted in taxonomic assignments which were considered spurious. This reflects limitations in public databases, and in particular, the paucity of *V. corneae* genomes in such databases.

## Conclusion

As deep sequencing is becoming increasingly available across health systems, its application is expected to improve individual case management but also the public health response to outbreaks and emerging infections. Further development and validation of mNGS are needed to overcome existing diagnostic and operational challenges associated with routine mNGS use.

## Note

This study has been presented in part at the 33rd European Congress of Clinical Microbiology and Infectious Diseases (ECCMID), Copenhagen, Denmark, April 2023.

## Supplementary Data

550000 Supplement

## References

[1] Ting DSJ Ho CS Deshmukh R Said DG Dua HS . Infectious keratitis: an update on epidemiology, causative microorganisms, risk factors, and antimicrobial resistance. Eye (Lond). 2021;35(4):1084-101. 10.1038/s41433-020-01339-3 33414529 PMC8102486

[2] Ibrahim YW Boase DL Cree IA . Factors affecting the epidemiology of Acanthamoeba keratitis. Ophthalmic Epidemiol. 2007;14(2):53-60. 10.1080/09286580600920281 17464851

[3] Ahmadikia K Aghaei Gharehbolagh S Fallah B Naeimi Eshkaleti M Malekifar P Rahsepar S Distribution, prevalence, and causative agents of fungal keratitis: a systematic review and meta-analysis (1990 to 2020). Front Cell Infect Microbiol. 2021;11:698780. 10.3389/fcimb.2021.698780 34513726 PMC8428535

[4] Martin C Löw U Quintin A Schießl G Gärtner B Heim A Epidemic keratoconjunctivitis: efficacy of outbreak management. Graefes Arch Clin Exp Ophthalmol. 2022;260(1):173-80. 10.1007/s00417-021-05344-4 34406500 PMC8763748

[5] Han B Pan G Weiss LM . Microsporidiosis in Humans. Clin Microbiol Rev. 2021;34(4):e0001020. 10.1128/CMR.00010-20 34190570 PMC8404701

[6] Das S Sharma S Sahu SK Nayak SS Kar S . Diagnosis, clinical features and treatment outcome of microsporidial keratoconjunctivitis. Br J Ophthalmol. 2012;96(6):793-5. 10.1136/bjophthalmol-2011-301227 22437900

[7] Tan J Lee P Lai Y Hishamuddin P Tay J Tan AL Microsporidial keratoconjunctivitis after rugby tournament, Singapore. Emerg Infect Dis. 2013;19(9):1484-6. 10.3201/eid1909.121464 23965938 PMC3810903

[8] Liu C Chen Q Fu P Shi YY . Anncaliia algerae microsporidiosis diagnosed by metagenomic next-generation sequencing, China. Emerg Infect Dis. 2022;28(7):1466-70. 10.3201/eid2807.212315 35731183 PMC9239868

[9] Health Ministry suspects parasite in Sea of Galilee causing eye infections. Jerusalem: The Times of Israel; 8 Nov 2022. Available from: https://www.timesofisrael.com/health-ministry-suspects-parasite-in-sea-of-galilee-causing-eye-infections

[10] Lubitz Greenbaum I, Barequet I, Cohen G, Ninio S, Sukenik A, Gihon I, et al. In the public eye - the story of an elusive aquatic predator. Annual meeting of the Israel Society for Parasitology, Protozoology and Tropical Diseases, Ramat Gan, Israel, March 2023.

[11] Fan NW Wu CC Chen TL Yu WK Chen CP Lee SM Microsporidial keratitis in patients with hot springs exposure. J Clin Microbiol. 2012;50(2):414-8. 10.1128/JCM.05007-11 22116156 PMC3264156

[12] Wang WY Chu HS Lin PC Lee TF Kuo KT Hsueh PR Outbreak of microsporidial keratoconjunctivitis associated with water contamination in swimming pools in Taiwan. Am J Ophthalmol. 2018;194:101-9. 10.1016/j.ajo.2018.07.019 30055152

[13] Sanpool O Thanathanee O Laummuanwai P Maleewong W Intapan PM . Molecular identification of microsporidian species in patients with epithelial keratitis. J Med Microbiol. 2020;69(3):414-8. 10.1099/jmm.0.001164 32011230

[14] Jayahar Bharathi M Murugan N Ramesh Kumar G Ramakrishnan R Anitha V Ramesh S . Vittaforma corneae keratitis in southern India: role of a novel duplex PCR. J Med Microbiol. 2013;62(Pt 4):553-9. 10.1099/jmm.0.051722-0 23319308

[15] Chiu CY Miller SA . Clinical metagenomics. Nat Rev Genet. 2019;20(6):341-55. 10.1038/s41576-019-0113-7 30918369 PMC6858796

[16] Gonzales JA Doan T VanZante A Stewart JM Sura A Reddy A Detection of Tropheryma whipplei genome from the aqueous humor by metagenomic sequencing. Ann Intern Med. 2021;174(9):1329-30. 10.7326/L20-1470 34125575

[17] Zhu J Xia H Tang R Ng TK Yao F Liao X Metagenomic next-generation sequencing detects pathogens in endophthalmitis patients. Retina. 2022;42(5):992-1000. 10.1097/IAE.0000000000003406 35019890

[18] Low L Nakamichi K Akileswaran L Lee CS Lee AY Moussa G Deep metagenomic sequencing for endophthalmitis pathogen detection using a nanopore platform. Am J Ophthalmol. 2022;242:243-51. 10.1016/j.ajo.2022.05.022 35660421 PMC9850836

[19] Valdes L Bispo P Sobrin L . Application of metagenomic sequencing in the diagnosis of infectious uveitis. Semin Ophthalmol. 2020;35(5-6):276-9. 10.1080/08820538.2020.1818795 33073643

[20] Seitzman GD Hinterwirth A Zhong L Cummings S Chen C Driver TH Metagenomic deep sequencing for the diagnosis of corneal and external disease infections. Ophthalmology. 2019;126(12):1724-6. 10.1016/j.ophtha.2019.06.013 31421897

[21] Bountogo M Sié A Coulibaly B Ruder K Chen C Zhong L Deep sequencing analysis of acute conjunctivitis in Burkina Faso, Africa. Int Health. 2023;15(1):101-3. 10.1093/inthealth/ihac001 35076074 PMC9808514

[22] Lalitha P Prajna NV Sikha M Gunasekaran R Hinterwirth A Worden L Evaluation of metagenomic deep sequencing as a diagnostic test for infectious keratitis. Ophthalmology. 2021;128(3):473-5. 10.1016/j.ophtha.2020.07.030 32682834 PMC7856230

[23] Redd TK Lalitha P Prajna NV Sikha M Gunasekaran R Hinterwirth A Impact of sample collection order on the diagnostic performance of metagenomic deep sequencing for infectious Keratitis. Cornea. 2022;41(1):39-44. 10.1097/ICO.0000000000002766 34870622 PMC8649208

[24] Ung L Bispo PJM Doan T Van Gelder RN Gilmore MS Lietman T Clinical metagenomics for infectious corneal ulcers: Rags to riches? Ocul Surf. 2020;18(1):1-12. 10.1016/j.jtos.2019.10.007 31669750 PMC9837861

[25] Chen JS Hsu TK Hsu BM Chao SC Huang TY Ji DD Swimming pool-associated Vittaforma-like microsporidia linked to microsporidial keratoconjunctivitis outbreak, Taiwan. Emerg Infect Dis. 2019;25(11):2100-3. 10.3201/eid2511.181483 31625849 PMC6810191

[26] Chen JS Hsu BM Tsai HC Chen YP Huang TY Li KY Molecular surveillance of Vittaforma-like microsporidia by a small-volume procedure in drinking water source in Taiwan: evidence for diverse and emergent pathogens. Environ Sci Pollut Res Int. 2018;25(19):18823-37. 10.1007/s11356-018-2081-4 29713979

[27] Kwok AKH Tong JMK Tang BSF Poon RWS Li WWT Yuen KY . Outbreak of microsporidial keratoconjunctivitis with rugby sport due to soil exposure. Eye (Lond). 2013;27(6):747-54. 10.1038/eye.2013.55 23598669 PMC3682360

