# Supplementary material for: Metagenomic sequencing for investigation of a national keratoconjunctivitis outbreak, Israel, 2022
Source: Euro Surveill. 2023 Aug 3;28(31):2300010. doi: 10.2807/1560-7917.ES.2023.28.31.2300010 (PMC10401915; doi:10.2807/1560-7917.ES.2023.28.31.2300010)
Supplement: Supplement [file 23-00010_MORAN-GILLAD_Supplement.pdf]

Supplementary materials

This supplementary material is hosted by *Eurosurveillance* as supporting information alongside the article [**Metagenomic sequencing for investigation of a national keratoconjunctivitis outbreak**], on behalf of the authors, who remain responsible for the accuracy and appropriateness of the content. The same standards for ethics, copyright, attributions and permissions as for the article apply. Supplements are not edited by *Eurosurveillance* and the journal is not responsible for the maintenance of any links or email addresses provided therein

Table S1: Taxonomic assignments of mNGS reads using kraken2 with the Standard-8 database.

Taxonomic assignment	taxid	Number of reads	Proportion (%)
Eukaryota	2759	111,155,869	86.12
Bacteria	2	11,903	0.01
Viruses	10239	6,357	0
Archaea	2157	183	0
unclassified		17,899,998	13.87
**Total (fwd and rev)**		**129,074,310**	**100.00**

Table S2: The most abundant taxonomic assignments of host (human)-removed mNGS reads using kraken2 with the Virus database

**Taxonomic assignment**	**taxid**	**Number of reads**	**Proportion of total reads (%)**
BeAn 58058 virus	67082	13,419	0.36
Human endogenous retrovirus K	45617	3,568	0.1
Salmonella phage vB_SenS_BPS1	2801546	1,288	0.03
Tequatrovirus kaw	2844208	1,246	0.03
Simbu orthobunyavirus	35306	1,056	0.03
Choristoneura fumiferana granulovirus	56947	858	0.02
Others	---	202	0.01
Unclassified	---	3,664,671	99.41
**Total**	**---**	**3,686,308**	**100**

Table S3: The most abundant taxonomic assignments of host (human)-removed mNGS reads using kraken2 with the EuPathDB46 database

**Taxonomic assignment**	**taxid**	**Number of reads**	**Proportion of Total reads (%)**	**Proportion of Assigned reads (%)**
Vittaforma corneae	**42399**	**34,665**	**0.94**	**96.57**
Enterocytozoon bieneusi	31281	156	0.004	0.44
Enterospora canceri	1081671	89	0.002	0.25
Nematocida displodere	1805483	84	0.002	0.23
Nosema ceranae	40302	67	0.002	0.19
Others	---	834	0.023	2.32
Unclassified	---	3,650,413	99.026	
**Total**	**---**	**3,686,308**	**100**	

Table S4: Top matching megaBLAST results for deconvoluted amplicons derived from a PCR-positive environmental sample.

Amplicon	Taxon	Total score	Query coverage (%)	E-value	Identity (%)	Accession
**env-Alt1-amp**	*Vittaforma corneae*	370	100	5.00E-98	96.43	MH333251.1
**env-Alt2-amp**	*Microsporidium* sp.	387	100	5.00E-103	97.77	MF374898.1

Table S5: Taxonomic assignments of long sequence reads at domain/kingdom level using kraken2 with the Standard-8 database.

**Taxonomic assignment**	**taxid**	**Number of reads**	**Proportion (%)**
Bacteria	2	439,688	30.78
Eukaryota	2759	67,335	4.71
Archaea	2157	2,693	0.19
Viruses	10239	384	0.03
Unclassified		918,464	64.29
**Total reads**		**1,428,564**	**100**

Table S6: Taxonomic assignments at the phylum level of long reads using kraken2 with the EuPathDB46 database

**Taxonomic assignment**	**taxid**	**Number of reads**	**Proportion (%)**
Ascomycota	4890	5,459	0.38
Discosea	555280	1,943	0.14
Apicomplexa	5794	1,874	0.13
Euglenozoa	33682	915	0.06
Basidiomycota	5204	838	0.06
Mucoromycota	1913637	393	0.03
Blastocladiomycota	451459	280	0.02
**Microsporidia**	**6029**	**117**	**0.01**
Evosea	2605435	77	0.01
Chytridiomycota	4761	63	0
Heterolobosea	5752	33	0
Unclassified		1,416,572	99.16
**Total reads**		**1,428,564**	**100**

Table S7: Taxonomic assignments at the species level of long reads assigned to the phylum Microsporidia using kraken2 with the EuPathDB46 database

Taxonomic assignment	taxid	Number of reads
Mitosporidium daphniae	1485682	22
Amphiamblys sp. WSBS2006	1866961	18
Hamiltosporidium tvaerminnensis	1176355	8
Anncaliia algerae	723287	6
Pseudoloma neurophilia	146866	5
Vavraia culicis	103449	4
Hepatospora eriocheir	1081669	3
Trachipleistophora hominis	72359	3
Vittaforma corneae	**42399**	**3**
Ordospora colligata	174685	3
Spraguea lophii	51541	3
Nosema ceranae	40302	3
Nosema bombycis	27978	3
Edhazardia aedis	70536	2
Enterocytozoon hepatopenaei	646526	2
Enterospora canceri	1081671	2
Nematocida displodere	1805483	2

Encephalitozoon cuniculi	6035	1
Enterocytozoon bieneusi	31281	1
Encephalitozoon intestinalis	58839	1
**Total reads**		**1,428,564**

Table S8: Taxonomic assignments of long sequence reads using kraken2 with the Virus database

Taxonomic assignment	taxid	Number of reads	Proportion (%)
Lambdavirus lambda	10710	10,272	0.72
Lambdavirus HK630	2169969	589	0.04
Lambdavirus HK629	2169968	367	0.03
Eptesicus fuscus gammaherpesvirus	2035399	267	0.02
Escherichia virus DE3	2169967	221	0.02
Pandoravirus salinus	1349410	179	0.01
Pandoravirus neocaledonia	2107708	141	0.01
Pandoravirus inopinatum	1605721	102	0.01
Polybotosvirus Atuph07	2560286	101	0.01
Pandoravirus macleodensis	2107707	96	0.01
Choristoneura fumiferana granulovirus	56947	92	0.01
Erskinevirus asesino	2169882	88	0.01
Pandoravirus dulcis	1349409	74	0.01
Efbeekayvirus P184	2955844	71	0.01
Synechococcus phage S-SRM01	2781608	71	0.01
Pandoravirus quercus	2107709	70	0.01
Pseudomonas phage YMC12/01/R24	2283028	61	0
Bertelyvirus BL9	2734192	57	0

Megavirus chiliensis	1094892	55	0
Menderavirus mendera	2846389	53	0
Moonbeamvirus moonbeam	2560334	52	0
Haifavirus tim68	2955972	52	0
Heterosigma akashiwo virus 01	97195	48	0
Synechococcus phage S-SZBM1	2926475	46	0
Spodoptera exigua multiple nucleopolyhedrovirus	10454	46	0
Busanvirus ACP17	2560261	45	0
Bronvirus silverleaf	2955544	45	0
Others (<1% of assigned reads)		2614	0.18
Unclassified		1,412,589	98.88
**Total reads**		**1,428,564**	**100**

Table S9: Taxonomic assignments of long sequence reads using kraken2 with the Standard-8 database

Taxonomic assignment	taxid	Number of reads	Proportion (%)
Homo sapiens	9606	67,297	4.71
Escherichia coli	562	17,114	1.2
Klebsiella quasipneumoniae	1463165	1,660	0.12
Sulfuritalea hydrogenivorans	748811	1,326	0.09
Aeromonas veronii	654	1,318	0.09
Rubrivivax gelatinosus	28068	1,159	0.08
Rhizobacter sp. AJA081-3	2753607	1,085	0.08
Ramlibacter tataouinensis	94132	1,013	0.07
Sulfuricaulis limicola	1620215	996	0.07
Rheinheimera sp. F8	1763998	995	0.07
Luteolibacter sp. SL250	2995170	917	0.06
Inhella inkyongensis	392593	894	0.06
Aquincola tertiaricarbonis	391953	756	0.05
Limnobacter sp. SAORIC-580	2732163	747	0.05
Variovorax paradoxus	34073	739	0.05
Luteolibacter ambystomatis	2824561	703	0.05
Salmonella enterica	28901	630	0.05
Kinneretia sp. DAIF2	2714952	629	0.04

Rheinheimera sp. MM224	3019969	623	0.04
Pseudomonas sp. SL4(2022)	2994661	610	0.04
Sphaerotilus sp. FB-5	2914710	610	0.04
Limnohabitans sp. 63ED37-2	1678128	591	0.04
Lacibacter sp. S13-6-22	2760713	565	0.04
Leptothrix cholodnii	34029	562	0.04
Pseudomonas alcaligenes	43263	562	0.04
Citrobacter portucalensis	1639133	561	0.04
Lacunisphaera limnophila	1838286	518	0.04
Flavobacterium ammoniigenes	1751095	517	0.04
Aquabacterium sp. J223	2898431	506	0.04
Caldimonas thermodepolymerans	215580	480	0.03
Azospira restricta	404405	473	0.03
Curvibacter sp. AEP1-3	1844971	471	0.03
Sorangium cellulosum	56	454	0.03
Hydrogenophaga sp. SL48	2806347	446	0.03
Rheinheimera mangrovi	2498451	444	0.03
Cupriavidus taiwanensis	164546	438	0.03
Usitatibacter rugosus	2732067	437	0.03
Usitatibacter palustris	2732487	421	0.03

Qipengyuania soli	2782568	419	0.03
Sphaerotilus sulfidivorans	639200	407	0.03
Ilumatobacter coccineus	467094	402	0.03
Stenotrophomonas maltophilia	40324	401	0.03
Niveibacterium microcysteis	2811415	401	0.03
Urbifossiella limnaea	2528023	399	0.03
Tsuneonella dongtanensis	692370	390	0.03
Candidatus Fonsibacter ubiquis	1925548	376	0.03
Cupriavidus pauculus	82633	370	0.03
Anaeromyxobacter sp. Fw109-5	404589	362	0.03
Ideonella dechloratans	36863	362	0.03
Cylindrospermopsis raciborskii	77022	359	0.03
Luteitalea sp. TBR-22	2802971	356	0.03
Variovorax sp. HW608	1034889	352	0.03
Ferribacterium limneticum	76259	350	0.03
Anaeromyxobacter oryzae	2918170	349	0.02
Shewanella oneidensis	70863	346	0.02
Opitutus sp. GAS368	1882749	345	0.02

Chitinimonas koreensis	356302	345	0.02
Sandaracinus amylolyticus	927083	343	0.02
Pseudomonas putida	303	335	0.02
Streptomyces venezuelae	54571	333	0.02
Luteolibacter luteus	2728835	327	0.02
Erythrobacter sp. SDW2	2907154	323	0.02
Microcystis aeruginosa	1126	323	0.02
Hydrogenophaga taeniospiralis	65656	322	0.02
Rhizobacter gummiphilus	946333	317	0.02
Others (<1% of assigned reads)		8,437	0.6
Unclassified		1,300,146	91.01
**Total reads**		**1,428,564**	**100**

Table S10: Strain-level comparison of host-removed short reads from the clinical metagenome to either *V. corneae* ATCC 50505 genome or an environmental 6kb *V. corneae* long read.

Variable	Contig JH370142.1 of ATCC 50505	Environmental 6kb long read
Total length	30,001	6,671
Bases covered by reads	2,239	6,598
Bases of total mapped reads	59,129	399,642
Breadth of coverage (%)	7.46%	98.91%
Mean depth of coverage	1.97	59.91

Figure S1. Number of *V. corneae* reads identified in publicly available Sea of Galilee MGx samples. Number of *V. corneae* reads identified in 61 publicly available Sea of Galilee MGx samples (from bioprojects PRJNA488159, PRJNA497963, and PRJNA637457) using kraken2 with the EuPathDB46 database.

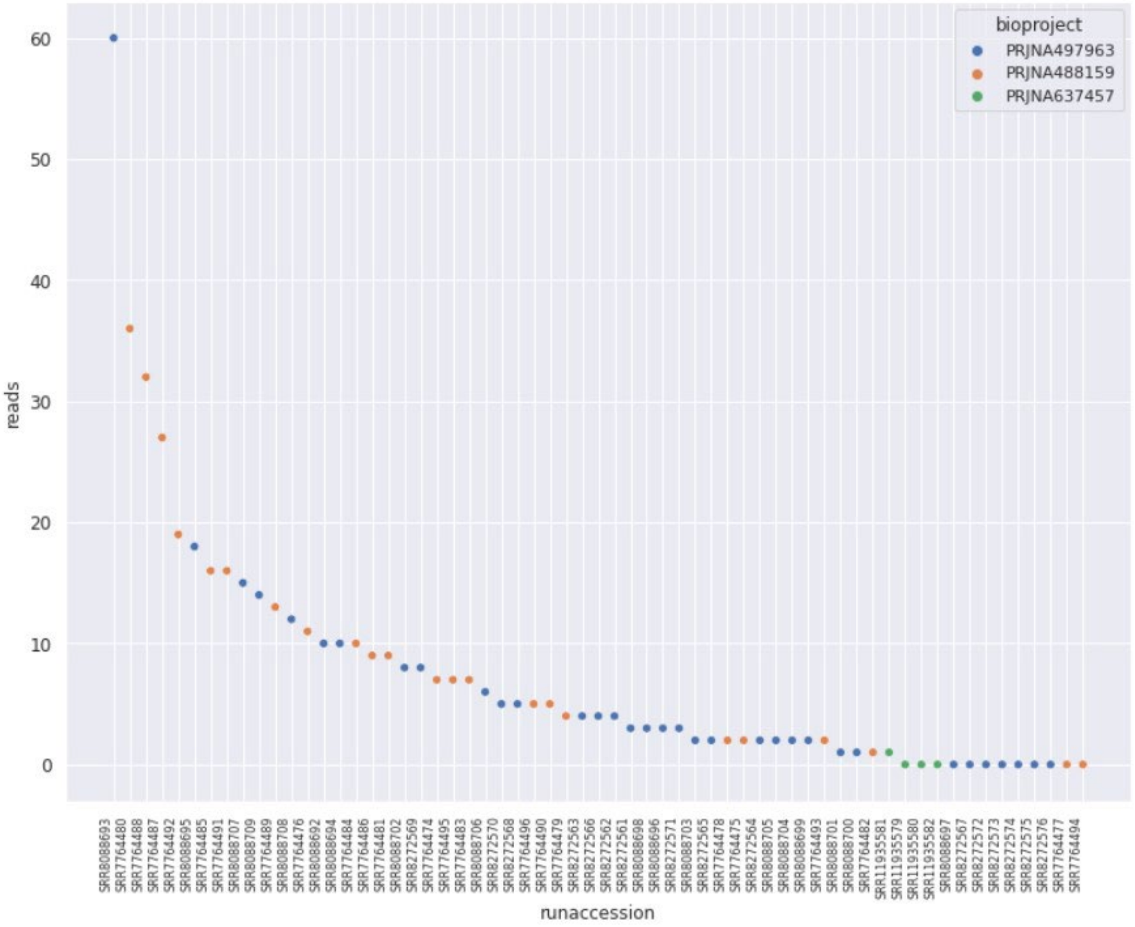

Figure S2. Detection and characterisation of *V. corneae* reads from historical samples.

For each of the top four abundant metagenomic samples (SRR8088693, SRR7764488, SRR7764487, and SRR7764492), mapping of the reads to the MAG derived from clinical sample, provided a consensus sequence of the respective SSU. When aligned to the *in silico* predicted real time PCR amplicons (including the isolated MAG, reference genome and environmental sample), regions of variation were observed for all historical samples.

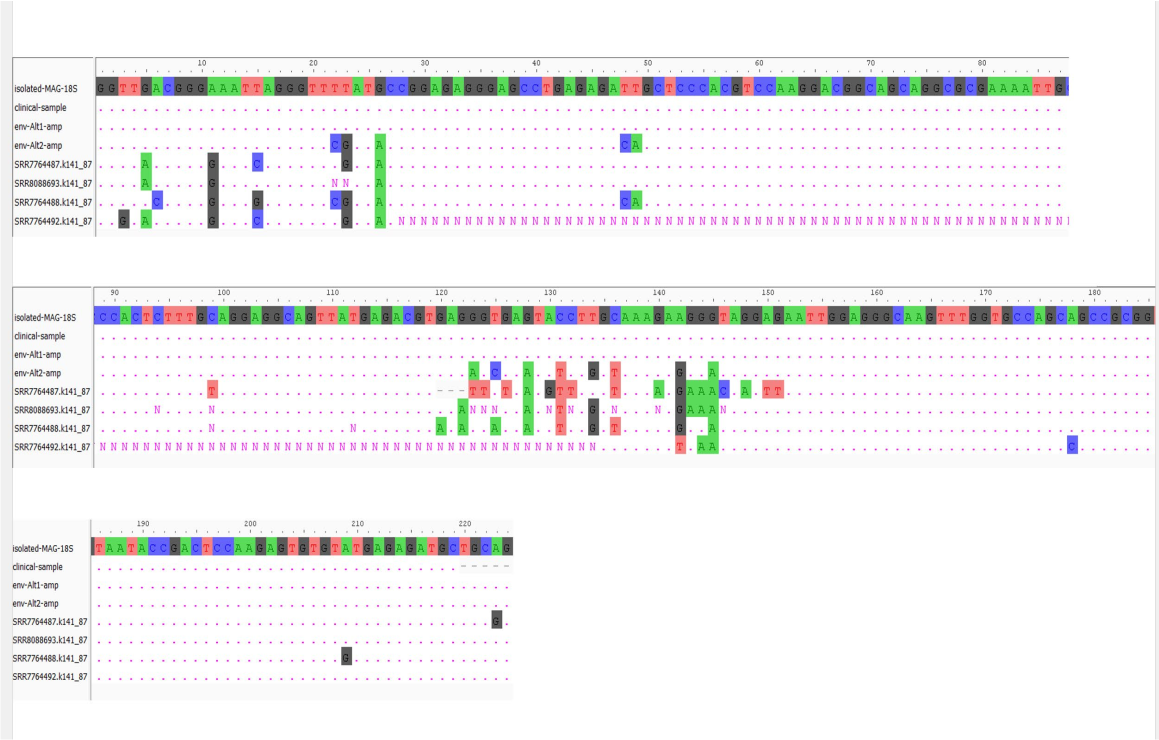

Supplementary protocol – real time PCR

MAG-derived PCR protocol for *Vittaforma corneae* detection:

Forward primer: 5'-GGTTGACGGGAAATTAGGGT-3'

Reverse primer: 5'-CTGCAGCATCTCTCATACACAC-3'

Reaction mix: 5.5 µl water, 1.0 µl primers, 12.5 µl mastermix for a total of 19.0 µl

Template DNA: 6 µl

Total reaction volume: 25 µl

PCR conditions: hold 96.5°c – 60 sec; denaturation 95°c – 6 sec, primer annealing 56°c – 8 sec, extension 72°c – 24 sec; number of cycles: 40

Analysis – expected amplicon size 224 bp, melting curve analysis – 85.76°c± 0.5°c

The PCR was performed on a Rotor-Gene instrument with negative controls using the KAPA SYBR FAST qPCR Master Mix (x2) (KAPABIOSYSTEMS, Boston, MA, USA).
